# Three-Dimensional, Image-Based Evaluation of the L5 Vertebral Body and Its Ossification Center in Human Fetuses

**DOI:** 10.3390/brainsci15111229

**Published:** 2025-11-15

**Authors:** Magdalena Grzonkowska, Michał Kułakowski, Karol Elster, Zofia Dzięcioł-Anikiej, Beata Zwierko, Sara Kierońska-Siwak, Magdalena Konieczna-Brazis, Michał Banasiak, Stanisław Orkisz, Mariusz Baumgart

**Affiliations:** 1 Department of Normal Anatomy, The Ludwik Rydygier Collegium Medicum in Bydgoszcz, The Nicolaus Copernicus University in Toruń, 87-100 Toruń, Poland; m.z.banasiak@gmail.com (M.B.); mariusz.baumgart@cm.umk.pl (M.B.); 2 Clinical Department of Orthopedics and Traumatology, Jan Biziel University Hospital nr 2 in Bydgoszcz, Nicolaus Copernicus University in Toruń, 87-100 Toruń, Poland; mkulakowski@poczta.fm (M.K.); karol.elster@gmail.com (K.E.); 3 Department of Rehabilitation, Medical University of Białystok, 15-089 Białystok, Poland; zofia.dzieciol-anikiej@umb.edu.pl; 4 Laboratory of Physical Activity, Clinical Research Center (CBK), Medical University of Białystok, 15-089 Białystok, Poland; 5 Department of Electroradiology, Faculty of Health Sciences, Collegium Medicum in Bydgoszcz, Nicolaus Copernicus University in Toruń, 85-626 Bydgoszcz, Poland; beata.zwierko@cm.umk.pl; 6 Department of Clinical Pathomorphology, Faculty of Medicine, Collegium Medicum in Bydgoszcz, Nicolaus Copernicus University in Toruń, 85-094 Bydgoszcz, Poland; sara.kieronska@cm.umk.pl; 7 Department of Neurology and Clinical Neurophysiology, Jan Biziel University Hospital No. 2, Collegium Medicum in Bydgoszcz, Nicolaus Copernicus University in Torun, Ujejskiego Street 75, 85-168 Bydgoszcz, Poland; magda.brazis@cm.umk.pl; 8 Department of Normal and Clinical Anatomy, Faculty of Medicine, University of Social Sciences in Łódź, 90-113 Łódź, Poland; orkisz.stanislaw@gmail.com

**Keywords:** fetus, ossification center, vertebral body

## Abstract

**Objectives:** The aim of this study was to characterize the developmental trajectories of the fifth lumbar vertebra in human fetuses by assessing the growth of its vertebral body and ossification center using linear, planar, and volumetric measurements. **Methods**: A total of 54 human fetuses (26 male and 28 female) aged 17–30 weeks of gestation were examined. Computed tomography, digital image analysis, 3D reconstruction, and statistical modeling were used to quantify morphometric parameters of the L5 vertebral body and its ossification center. **Results**: All measured parameters demonstrated consistent age-related growth following a linear pattern. No statistically significant differences between sexes were observed in any measured diameter of the L5 vertebra or its ossification center within the examined gestational age range. **Conclusions**: The normative morphometric data and growth curves obtained for the L5 vertebra and its ossification center provide age-specific reference values that may aid in prenatal diagnostics. These findings can support clinicians in estimating gestational age, assessing vertebral development on ultrasound, and detecting congenital spinal anomalies and skeletal dysplasias at an early stage. Further multicenter studies including a broader gestational age range are warranted to strengthen the generalizability and clinical applicability of these results.

## 1. Introduction

The development of the fetal skeletal system follows a spatially and temporally coordinated pattern, in which the appearance and growth of ossification centers reflect the degree of mineralization and skeletal maturation. Quantitative morphometric assessment of these centers—including linear, surface, and volumetric parameters provides reliable indicators of fetal skeletal development and supports the estimation of gestational age, the detection of ossification delay, and the diagnosis of congenital skeletal dysplasias [1,2,3].

Each vertebra develops from three primary ossification centers—one in the vertebral body and two in the neural arches, followed later by secondary centers associated with the spinous and transverse processes, and along the superior and inferior margins of the vertebral body [4,5]. Ossification typically begins in the lower thoracic and upper lumbar regions and progresses in a craniocaudal direction [5,6].

Recent advances in high-resolution volumetric imaging, particularly computed tomography and micro–CT, have enabled precise three-dimensional reconstruction and quantitative assessment of fetal bone structures, facilitating the development of accurate growth models that surpass the capabilities of traditional autopsy-based methods [7,8,9,10,11,12,13].

Quantitative morphometric analyses of fetal lumbar vertebrae based on CT imaging have thus far been limited to the L3 and L4 levels [14,15]. However, the fifth lumbar vertebra occupies a unique anatomical and functional position within the lumbosacral junction. It serves as the transitional segment between the mobile lumbar spine and the fixed sacrum, transmitting considerable axial load and contributing to the biomechanics and curvature of the lower spine. Biomechanically, this junction represents the primary interface through which spinal loads are transferred to the pelvis and lower limbs, and it is therefore subjected to the highest degree of mechanical stress within the vertebral column [16]. Because of this transitional nature, L5 is frequently involved in congenital and developmental anomalies such as caudal regression syndrome, spina bifida, and lumbosacral dysplasia [13].

From a diagnostic standpoint, the evaluation of L5 ossification is particularly relevant in prenatal imaging, as alterations in its morphology or developmental timing may reflect disturbances in the caudal progression of vertebral ossification or early defects in neural tube closure. These morphometric measurements are clinically important for identifying congenital and developmental abnormalities of the spine, which can significantly affect neural structures, including the spinal cord and nerve roots. Despite this clinical significance, no quantitative reference data have yet been established for the morphometric growth of the L5 vertebral body during fetal life.

Therefore, within the framework of translational neuroanatomy, the present study aimed to perform a comprehensive morphometric analysis of the L5 vertebral body and its ossification center in human fetuses. The objectives were:

- To quantify linear, surface, and volumetric parameters across different gestational ages and establish reference values;
- To assess potential sex-related differences;
- To determine the growth dynamics and develop mathematical models that best describe the observed relationships.

## 2. Materials and Methods

### 2.1. Examined Sample

The study material consisted of 55 human fetuses (27 males and 28 females) between the 17th and 30th weeks of gestation. The specimens were part of the collection of the Department of Normal Anatomy, Ludwik Rydygier Collegium Medicum in Bydgoszcz, Nicolaus Copernicus University in Toruń, Poland. Ethical approval for the study was obtained from the Bioethics Committee of Ludwik Rydygier Collegium Medicum in Bydgoszcz (Decision No. KB 275/2011, issued on 10 May 2011). All procedures were conducted in accordance with applicable legal regulations and the institutional Body Donation Program for adults and fetuses, in full compliance with the principles of the Declaration of Helsinki.

Morphometric analyses were performed at the Department of Anatomy, Ludwik Rydygier Collegium Medicum, Nicolaus Copernicus University in Toruń. Only fetuses with normal anatomical structure, preserved spinal integrity, and complete clinical documentation were included in the study. Exclusion criteria comprised congenital malformations, evidence of intrauterine growth restriction, spinal deformities, and other significant musculoskeletal pathologies that could affect the ossification process.

Gestational age was determined based on crown–rump length (CRL) and the date of the mother’s last menstrual period. Only cases demonstrating high concordance between both methods (correlation coefficient R = 0.98; *p* < 0.001) were included in the final analysis. This approach ensured accurate gestational age estimation and homogeneity of the study group. A detailed description of the study material, including its distribution by gestational age and sex, is presented in Table 1.

### 2.2. Morphometric Measurements

**Figure 1:**
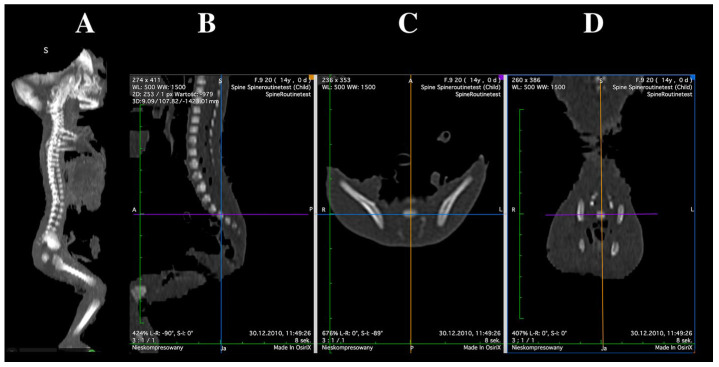
CT scan of a female fetus at 24 weeks of gestation: sagittal projection (**A**) and multiplanar reconstructions (MPRs) of the L5 vertebra in sagittal (**B**), transverse (**C**), and frontal (**D**) planes, obtained using OsiriX 3.9.

Imaging examinations were performed using a Siemens Biograph mCT 128 computed tomography scanner (Siemens Healthcare GmbH, Erlangen, Germany) at the Department of Positron Emission Tomography and Molecular Imaging, Collegium Medicum, Nicolaus Copernicus University in Bydgoszcz. The acquired images were reconstructed in DICOM format, generating transverse sections with a slice thickness of 0.4 mm (Figure 1).

The range of grayscale values in Hounsfield units (HU) extended from −275 to −134 for minimum values and from +1165 to +1558 for maximum values, corresponding to a window width (WW) of 1404–1692 HU and a window level (WL) of +463 to +712 HU. The detailed imaging protocol was as follows: tube current 60 mAs, tube voltage 80 kV, pitch 0.35, field of view (FOV) 180 mm, and rotation time 0.5 s. Images were acquired with a slice thickness of 0.4 mm, an increment of 0.6 mm, and reconstructed using a B45f medium kernel.

**Figure 2:**
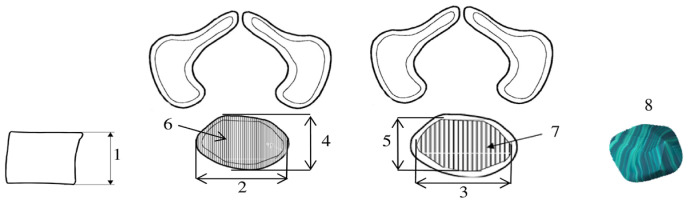
Schematic illustration of morphometric measurements of the L5 vertebral body and its ossification center.

Morphometric evaluation included both the vertebral body and the primary ossification center of the fifth lumbar vertebra. Measurements were performed according to a predefined protocol (Figure 2). For each fetus, linear diameters, cross-sectional areas, and volumes were assessed for the L5 vertebral body and its ossification center. Despite the persistence of cartilaginous tissues, the margins of ossifying structures were sufficiently distinct to allow precise and reproducible quantification of all morphometric parameters [2,17,18]. The contours of the ossification center and the vertebral body were defined semi-automatically, by manually outlining the regions of interest (ROI) on each CT slice with reference to density thresholds distinguishing bone from surrounding cartilage. The outlined ROIs were then used to calculate surface area and volume in OsiriX MD 3.9 software.

A total of eight morphometric parameters related to the L5 vertebral body and its primary ossification center were measured (Figure 2):

1. Vertebral body height—the maximum distance between the superior and inferior borders of the vertebral body in the sagittal plane;
2. Transverse diameter of vertebral body—the maximum distance between the lateral edges of the vertebral body in the transverse plane;
3. Transverse diameter of body ossification center—the maximum distance between the lateral edges of the ossification center in the transverse plane;
4. Sagittal diameter of vertebral body—the maximum distance between the anterior and posterior borders of the vertebral body in the sagittal plane;
5. Sagittal diameter of body ossification center—the maximum distance between the anterior and posterior borders of the ossification center in the sagittal plane;
6. Cross-sectional area of vertebral body—the area determined based on the contour of the vertebral body in the transverse plane;
7. Cross-sectional area of body ossification center—the area determined based on the contour of the ossification center in the transverse plane;
8. Volume of the ossification center—calculated using three-dimensional reconstruction software(OsiriX MD 3.9), taking into account spatial orientation and X-ray attenuation properties of the mineralizing tissue.

### 2.3. Statistical Analysis

Statistical analyses were performed using Statistica software (version 12.5; StatSoft, Tulsa, OK, USA) and PQStat software (version 1.6.2; PQStat Software, Poznań, Poland). The distribution of quantitative variables was assessed using the Shapiro–Wilk test, and the homogeneity of variances using Fisher’s test. Comparisons of morphometric parameters between sexes were conducted using the independent samples t-test. For variables meeting the assumptions of normality and homogeneity of variance, a one-way analysis of variance (ANOVA) was applied, followed by Tukey’s post hoc test to identify significant intergroup differences. When these assumptions were not met, the Kruskal–Wallis test was used as a nonparametric alternative.

The growth dynamics of the vertebral body and its ossification center were analyzed using linear and nonlinear regression models, and the model fit was evaluated using the coefficient of determination (R^2^). Relationships between quantitative variables were assessed with the Pearson correlation coefficient (r). A *p*-value < 0.05 was considered statistically significant.

Each measurement was performed three times under identical technical conditions, and the mean value was used for analysis. The reliability and repeatability of the measurement method were evaluated using the intraclass correlation coefficient (ICC), calculated from repeated measurements performed by a single observer (M.G.). The ICC values demonstrated excellent intra-observer consistency (*p* < 0.001), confirming high reproducibility of the applied morphometric protocol (Table 2).

## 3. Results

The mean values and standard deviations of the analyzed morphometric parameters of the L5 vertebral body and its ossification center in human fetuses during the studied developmental period are presented in Table 3 and Table 4. Statistical analysis revealed no significant sex-related differences in any of the measured parameters; therefore, a single unified growth curve was generated for each variable.

### 3.1. Morphometric Parameters of the L5 Vertebral Body

**Figure 3:**
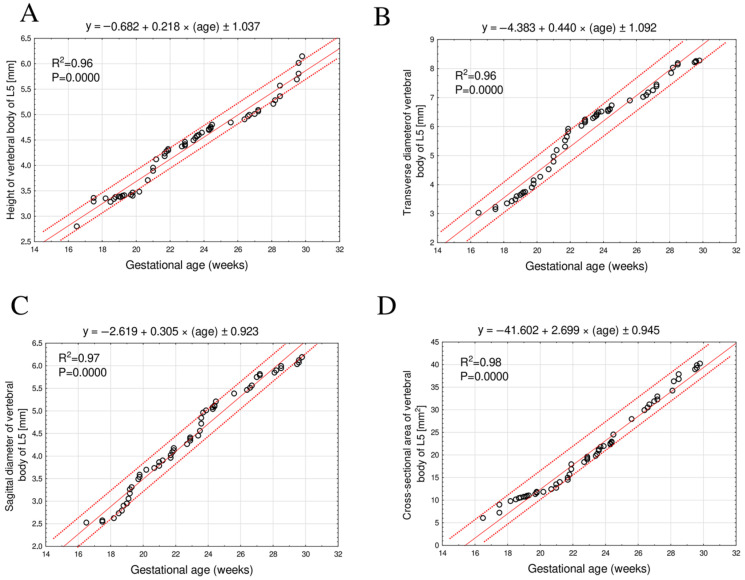
Regression models illustrating height (**A**), transverse diameter (**B**), sagittal diameter (**C**), and cross-sectional area (**D**) growth patterns of the L5 vertebral body as a function of gestational age.

The mean height of the L5 vertebral body increased linearly from 2.89 ± 0.03 mm at 17 weeks to 5.91 ± 0.20 mm at 30 weeks of gestation, following the equation: y = −0.682 + 0.218 × (age) ± 1.037; R^2^ = 0.96 (Figure 3A).

The mean transverse diameter of the L5 vertebral body during the same gestational period ranged from 3.02 ± 0.02 mm to 8.24 ± 0.03 mm, following a linear growth function: y = −4.383 + 0.440 × (age) ± 1.092; R^2^ = 0.96 (Figure 3B).

The mean sagittal diameter of the L5 vertebral body increased from 2.52 mm at 17 weeks to 6.10 ± 0.07 mm at 30 weeks of gestation, also exhibiting a linear growth relationship: y = −2.619 + 0.305 × (age) ± 0.923; R^2^ = 0.97 (Figure 3C).

The mean cross-sectional area of the L5 vertebral body increased from 6.04 mm^2^ at 17 weeks to 39.54 ± 0.58 mm^2^ at 30 weeks of gestation, fitting a linear model: y = −41.602 + 2.699 × (age) ± 0.945; R^2^ = 0.98 (Figure 3D).

### 3.2. Morphometric Parameters of the L5 Ossification Center

**Figure 4:**
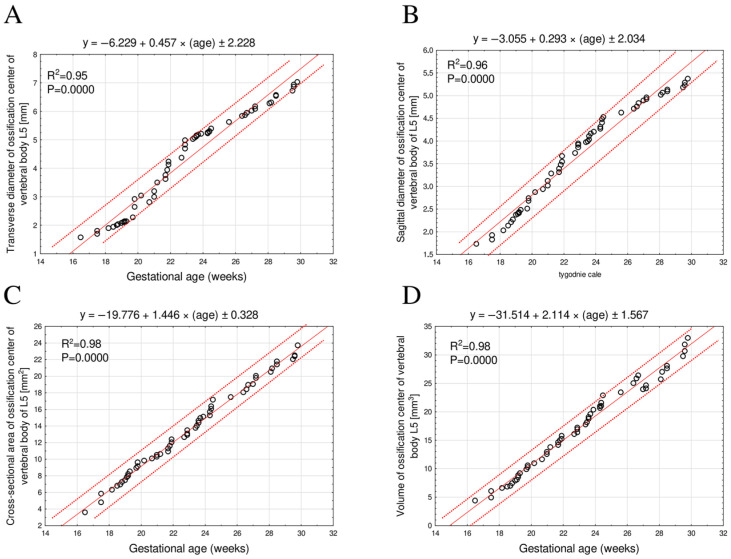
Regression models illustrating transverse diameter (**A**), sagittal diameter (**B**), cross-sectional area (**C**), and volume (**D**) growth patterns of the ossification center of the L5 vertebral body as a function of gestational age.

The mean transverse diameter of the L5 ossification center increased from 1.56 mm at 17 weeks of gestation to 6.88 ± 0.13 mm at 30 weeks, following a linear growth model described by the equation: y = −6.229 + 0.457 × (age) ± 2.228; R^2^ = 0.95 (Figure 4A).

The mean sagittal diameter of the L5 ossification center increased from 1.73 mm at 17 weeks to 5.27 ± 0.08 mm at 30 weeks of gestation, exhibiting a linear growth relationship: y = −3.055 + 0.293 × (age) ± 2.034; R^2^ = 0.96 (Figure 4B).

The mean cross-sectional area of the L5 ossification center increased from 3.60 mm^2^ at 17 weeks to 22.64 ± 0.73 mm^2^ at 30 weeks of gestation, fitting a linear growth model: y = −19.776 + 1.446 × (age) ± 0.328; R^2^ = 0.98 (Figure 4C).

The mean volume of the L5 ossification center increased from 4.36 mm^3^ at 17 weeks to 31.28 ± 1.42 mm^3^ at 30 weeks of gestation, also following a linear growth pattern: y = −31.514 + 2.114 × (age) ± 1.567; R^2^ = 0.98 (Figure 4D).

## 4. Discussion

The L5 vertebra plays a crucial role in transferring loads between the spine and the pelvis. Morphometric studies indicate that, during intrauterine development, the posterior elements of the lumbar vertebrae undergo substantial morphological transformation—from broad and robust structures to progressively slender and specialized bony components. These processes begin earliest in the L1 vertebra and latest in L5, with the most dynamic changes occurring between the 11th and 15th weeks of gestation. Such transformations are closely associated with microangiogenesis, progressive ossification, and the adaptation of the developing skeletal system to increasing mechanical loads. Understanding the dynamics of these developmental processes is essential for accurate interpretation of prenatal imaging studies, particularly in the diagnosis of spina bifida and other lumbosacral malformations. Both structural and functional abnormalities of the lumbar spine are frequent components of congenital malformation syndromes. Among the most common defects of this region is spina bifida, which may coexist with trisomy 21, 18, or 13. Developmental anomalies arising during the fetal period are typically detected prenatally or shortly after birth. Furthermore, these conditions are considered predisposing factors for the later development of low back pain syndromes in adulthood [19].

It is noteworthy that the L5 vertebra, positioned within the lumbosacral transitional segment, is subjected to particularly high mechanical loads and is the last lumbar vertebra to complete ossification [19]. According to observations by Sagi et al. [20], this delayed maturation may contribute to the increased prevalence of spondylolysis at the L4–L5 level. The findings of Czyż and Kędzia [19] further support this association, demonstrating that in the L5 vertebra, marked hypertrophy of the inferior articular processes may serve as a compensatory mechanism for the reduced mechanical strength of cartilaginous structures.

From an anatomical and clinical perspective, the L5 vertebra exhibits considerable morphological variability, as it constitutes part of the lumbosacral transitional zone. Lumbosacral transitional vertebrae (LSTV) are relatively common in the general population, with a reported prevalence ranging from 7% to 36%. These represent morphological variants of segmentation at the lumbosacral junction and are typically classified into two main types: sacralization of L5, in which the enlarged transverse processes fuse partially or completely with the sacrum or iliac bone, and lumbarization of S1, characterized by the presence of an additional intervertebral disc between the first and second sacral segments, effectively creating a “sixth” lumbar vertebra [5,21].

These anatomical variants are clinically significant, as they may influence the shape and dimensions of the L5 vertebral body and alter the biomechanics of the lumbosacral region. Sacralization may lead to nerve compression, increased pressure on surrounding soft tissues, and stretching of adjacent ligaments. These changes contribute to the development of degenerative spondylolisthesis, lumbar disc degeneration and herniation, as well as chronic low back pain. In females, sacralization may also cause difficulties during labor due to reduced pelvic mobility resulting from fusion at the lumbosacral joint [22].

A precise understanding of the normal morphometric development of L5 during the fetal period therefore provides a valuable reference point for interpreting such transitional anomalies in prenatal imaging. Furthermore, the quantitative models proposed in the present study may assist in identifying early deviations in ossification patterns that could underlie the later manifestation of lumbosacral transitional vertebrae or related biomechanical instabilities.

Szpinda et al. [23] conducted morphometric measurements of vertebral bodies in fetuses aged 17–30 weeks using computed tomography. They demonstrated that parameters such as vertebral body height increase up to the level of L2, remain relatively constant at L3–L4, and then gradually decrease toward the sacral region. A reduction in cross-sectional area and volume from L3 to L5 was also observed, indicating that the L5 vertebra is smaller than the superior lumbar vertebrae, which may account for its reduced mechanical strength. The present study confirmed this developmental tendency through a comparative analysis of morphometric parameters of adjacent vertebrae—L4 [14] and S1 [2]—in relation to L5.

In our study, all diameters of the L5 vertebral body and its ossification center demonstrated a proportional increase with advancing fetal age. Similar developmental trends were observed for both the S1 vertebral body and its ossification center. In fetuses aged 18–30 weeks, the linear growth of S1 vertebral body parameters was described by the following equations: height—y = −1.387 + 0.243 × age ± 0.075 (R^2^ = 0.93); transverse diameter—y = −4.032 + 0.389 × age ± 1.307 (R^2^ = 0.97); sagittal diameter—y = −2.432 + 0.279 × age ± 0.098 (R^2^ = 0.95); cross-sectional area—y = −36.860 + 2.346 × age ± 3.332 (R^2^ = 0.94). Likewise, the ossification center parameters of S1 were also age-dependent, increasing proportionally in a linear manner: transverse diameter—y = −4.596 + 0.361 × age ± 0.272 (R^2^ = 0.98); sagittal diameter—y = −3.292 + 0.270 × age ± 0.132 (R^2^ = 0.97); cross-sectional area—y = −24.302 + 1.473 × age ± 0.476 (R^2^ = 0.88); volume—y = −30.803 + 1.879 × age ± 3.328 (R^2^ = 0.90) [2].

Different growth patterns were observed for the L4 vertebra. The height, transverse, and sagittal diameters of the L4 vertebral body followed logarithmic growth functions: y = −11.797 + 5.208 × ln(age) ± 0.372 (R^2^ = 0.83); y = −23.462 + 9.428 × ln(age) ± 0.702 (R^2^ = 0.82); y = 2.770 + 13.521 × ln(age) ± 1.722 (R^2^ = 0.81). The cross-sectional area of the L4 vertebral body increased linearly (y = −30.683 + 1.976 × age ± 2.701; R^2^ = 0.87), whereas its volume followed a quadratic function (y = −93.983 + 0.385 × age^2^ ± 23.707; R^2^ = 0.88). Similarly, the transverse and sagittal diameters of the L4 vertebral body ossification center exhibited logarithmic growth: y = −27.106 + 10.178 × ln(age) ± 0.769 (R^2^ = 0.82) and y = −13.345 + 5.458 × ln(age) ± 0.424 (R^2^ = 0.81), respectively. In contrast, both the projected surface area and volume of the L4 ossification center increased linearly with fetal age: y = −30.683 + 1.976 × age ± 2.701 (R^2^ = 0.88) and y = −43.214 + 2.760 × age ± 4.085 (R^2^ = 0.86) [15].

Widaja et al. [3], using magnetic resonance imaging, demonstrated a linear increase in lumbar vertebral body dimensions, including both height and cross-sectional area. They also reported that the growth of the intervertebral spaces exceeds that of vertebral body height with advancing gestational age. This phenomenon is considered physiological and should not be misinterpreted as a pathological finding. Due to the limited sample size, their assessment of vertebral growth was conducted collectively for fetuses of both sexes. Similarly, in the present study, no statistically significant sex-related differences were observed in any of the analyzed parameters between 17 and 30 weeks of gestation.

Knowledge of the ossification sequence and morphometry of the L5 vertebra has considerable clinical importance. The normal timing of ossification center development is a key criterion for estimating gestational age and diagnosing skeletal disorders. Delayed ossification of L5 may indicate osteochondrodysplasia or hypophosphatasia—conditions characterized by impaired mineralization and abnormal vertebral morphology. Szpinda et al. [23] emphasized that congenital spinal anomalies such as caudal regression syndrome, diastematomyelia, and spina bifida often coexist with asymmetry of the vertebral arch ossification centers or incomplete fusion. Advances in modern imaging modalities, including ultrasound, computed tomography, and magnetic resonance imaging, now allow for detailed assessment of the shape, size, and localization of ossification centers. This facilitates the early detection of developmental abnormalities and supports appropriate prenatal management planning [24,25,26,27,28,29].

In prenatal ultrasound, CT, or MRI, deviations in the dimensions of the L5 vertebral body exceeding ±2 standard deviations (SD) from the predicted mean should be considered potentially abnormal. This statistical threshold aligns with general biometric principles, as approximately 95% of values in a normally distributed population fall within ±2 SD of the mean. Accordingly, measurements beyond this range—representing the outer ~5% of the population—may indicate developmental disturbances rather than physiological variability [30,31,32].

In practical terms, a z-score below –2 may suggest delayed ossification or impaired mineralization of the vertebral body, whereas a z-score above +2 could reflect disproportionate or accelerated growth. Although normative data for vertebral dimensions are still limited compared with long bone biometry, applying such statistical cut-offs can facilitate early recognition of atypical growth trajectories. Early identification of these deviations during prenatal imaging may aid in detecting skeletal dysplasias or early manifestations of the caudal regression spectrum before overt morphological abnormalities become evident.

Incorporating quantitative, z-score–based thresholds into prenatal spinal assessment protocols may therefore enhance the ability to distinguish physiological variation from pathological growth patterns and provide an objective framework for subsequent diagnostic management. Furthermore, the growth models established in this study could serve as a reference for future research and for the development of automated or AI-assisted systems capable of detecting deviations from normative morphometric standards.

### Limitations of the Study

A key limitation of the present study is the relatively narrow gestational age range of the analyzed specimens (17–30 weeks), which suggests that the proposed nomograms should be regarded as preliminary. Although the overall sample size was adequate, the distribution of fetuses across individual gestational weeks was uneven, which may have influenced the precision of the fitted growth models and the smoothness of the regression curves. This limitation should therefore be considered when interpreting week-to-week variability in the measured parameters.

A larger, multicenter investigation encompassing both earlier embryonic stages and the late third trimester, as well as including a broader ethnic representation, would strengthen the external validity of the findings. Furthermore, longitudinal correlations with postnatal imaging of newborns and infants would be valuable to determine whether early deviations from the proposed growth curves translate into clinically relevant postnatal abnormalities [2].

## 5. Conclusions

1. No sex-related differences were observed in any of the morphometric parameters of the L5 vertebral body or its ossification center.
2. All analyzed morphometric parameters of the L5 vertebral body and its ossification center increased proportionally with gestational age expressed in weeks.
3. The morphometric data obtained for the L5 vertebral body and its ossification center may serve as gestational age–dependent reference values, supporting fetal age assessment and ultrasound-based diagnosis of congenital anomalies. Further research on the growth patterns and morphometric characteristics of the L5 vertebra is warranted to deepen our understanding of its development and potential clinical significance.

## Figures and Tables

**Table 1:** Characteristics of the fetuses studied according to gestational age, crown–rump length, and sex.

Gestational Age	Crown-Rump Length (mm)				Number of Fetuses	Sex	
Weeks (Hbd-Life)	Mean	SD	Min.	Max.		♂	♀
17	115.00	-	115.00	115.00	1	0	1
18	133.33	5.77	130.00	140.00	3	1	2
19	149.50	3.82	143.00	154.00	8	3	5
20	161.00	2.71	159.00	165.00	4	2	2
21	174.75	2.87	171.00	178.00	4	3	1
22	185.00	1.41	183.00	186.00	4	1	3
23	197.60	2.61	195.00	202.00	5	2	3
24	208.67	3.81	204.00	213.00	9	5	4
25	214.00	-	214.00	214.00	1	0	1
26	229.00	5.66	225.00	233.00	2	1	1
27	237.50	3.33	233.00	241.00	6	6	0
28	249.50	0.71	249.00	250.00	2	0	2
29	253.00	0.00	253.00	253.00	2	0	2
30	263.25	1.26	262.00	265.00	4	3	1
Total					55	27	28

**Table 2:** Intra-class correlation coefficient (ICC) values for intra-observer repeatability.

Parameters of the L5 Vertebral Body	ICC
Height	0.994 *
Transverse diameter	0.994 *
Sagittal diameter	0.996 *
Cross-sectional area	0.997 *
**Parameters of the ossification center of the L5 vertebral body**	
Transverse diameter	0.996 *
Sagittal diameter	0.997 *
Cross-sectional area	0.997
Volume	0.9937

Intra-class correlation coefficients marked with * are statistically significant at *p*  <  0.0001.

**Table 3:** Morphometric parameters of the L5 vertebral body: height, transverse and sagittal diameters, cross-sectional area, and volume.

Gestational Age (Weeks)	N	Vertebral Body L5							
		Height (mm)		Transverse Diameter (mm)		Sagittal Diameter (mm)		Cross-Sectional Area (mm^2^)	
		Mean	SD	Mean	SD	Mean	SD	Mean	SD
17	1	2.80		3.02		2.52		6.04	
18	3	3.33	0.04	3.24	0.10	2.58	0.04	8.66	1.30
19	8	3.37	0.04	3.63	0.12	3.02	0.22	10.67	0.29
20	4	3.44	0.04	4.08	0.16	3.57	0.09	11.62	0.25
21	4	3.92	0.17	4.86	0.28	3.82	0.08	13.12	0.70
22	5	4.26	0.05	5.64	0.24	4.07	0.08	15.94	1.42
23	5	4.42	0.05	6.17	0.10	4.37	0.07	19.21	0.52
24	9	4.65	0.08	6.49	0.10	4.94	0.19	21.82	0.91
25	1	4.80		6.72		5.20		24.46	
26	2	4.87	0.04	6.96	0.09	5.42	0.06	28.86	1.40
27	5	5.02	0.05	7.26	0.15	5.68	0.14	31.73	0.97
28	2	5.25	0.05	7.93	0.13	5.87	0.04	35.20	1.46
29	2	5.47	0.15	8.16	0.04	5.97	0.04	37.32	0.76
30	4	5.91	0.20	8.24	0.03	6.10	0.07	39.54	0.58

**Table 4:** Morphometric parameters of the L5 vertebral body ossification center: transverse and sagittal diameters, cross-sectional area, and volume.

Gestational Age (Weeks)	N	Ossification Center L5							
		Transverse Diameter (mm)		Sagittal Diameter (mm)		Cross-Sectional Area (mm^2^)		Volume (mm^3^)	
		Mean	SD	Mean	SD	Mean	SD	Mean	SD
17	1	1.56		1.73		3.60		4.36	
18	3	1.79	0.10	1.93	0.10	5.64	0.77	5.84	0.84
19	8	2.06	0.07	2.34	0.12	7.62	0.62	7.96	0.85
20	4	2.71	0.34	2.70	0.15	9.39	0.41	10.38	0.45
21	4	3.12	0.29	3.09	0.15	10.36	0.25	12.73	0.86
22	5	3.93	0.26	3.47	0.14	11.64	0.57	14.94	0.59
23	5	4.77	0.26	3.88	0.09	13.16	0.44	16.86	0.66
24	9	5.19	0.08	4.22	0.16	15.16	0.77	20.00	1.19
25	1	5.39		4.53		17.13		22.89	
26	2	5.73	0.15	4.67	0.06	17.76	0.39	24.20	1.11
27	5	6.01	0.12	4.87	0.08	19.23	0.64	24.97	1.06
28	2	6.29	0.03	5.04	0.04	20.73	0.30	26.33	0.88
29	2	6.55	0.03	5.11	0.03	21.56	0.27	27.81	0.32
30	4	6.88	0.13	5.27	0.08	22.64	0.73	31.28	1.42

## Data Availability

Any additional data supporting this study are available from the corresponding author (M.G.) upon reasonable request due to (privacy and ethical restrictions).
